# On new inequalities of Hermite–Hadamard–Fejer type for harmonically convex functions via fractional integrals

**DOI:** 10.1186/s40064-016-2215-4

**Published:** 2016-05-17

**Authors:** Mehmet Kunt, İmdat İşcan, Nazlı Yazıcı, Uğur Gözütok

**Affiliations:** Department of Mathematics, Faculty of Sciences, Karadeniz Technical University, Trabzon, Turkey; Department of Mathematics, Faculty of Sciences and Arts, Giresun University, Giresun, Turkey

**Keywords:** Hermite–Hadamard inequality, Hermite–Hadamard–Fejer inequality, Riemann–Liouville fractional integrals, Harmonically convex functions, 26A51, 26A33, 26D10

## Abstract

In this paper, firstly, new Hermite–Hadamard type inequalities for harmonically convex functions in fractional integral forms are given. Secondly, Hermite–Hadamard–Fejer inequalities for harmonically convex functions in fractional integral forms are built. Finally, an integral identity and some Hermite–Hadamard–Fejer type integral inequalities for harmonically convex functions in fractional integral forms are obtained. Some results presented here provide extensions of others given in earlier works.

## Background

Let $f:I\subset \mathbb {R\rightarrow R}$ be a convex function defined on the interval *I* of real numbers and $a,b\in I$. The inequality

1 $$f\left( \frac{a+b}{2}\right) \le \frac{1}{b-a}\int _{a}^{b}f(x)dx\le \frac{ f(a)+f(b)}{2}$$

is well known in the literature as Hermite–Hadamard’s inequality (Hadamard 1893; Hermite 1883).

The most well-known inequalities related to the integral mean of a convex function *f* are the Hermite Hadamard inequalities or their weighted versions, the so-called Hermite–Hadamard-Fejér inequalities.

 Fejér (1906) established the following Fejér inequality which is the weighted generalization of Hermite–Hadamard inequality (1):

### **Theorem 1**

*Let*$f:\left[ a,b\right] \mathbb {\rightarrow R}$*be a convex function. Then the inequality*

2 $$\begin{aligned} f\left( \frac{a+b}{2}\right) \int _{a}^{b}g(x)dx\le \int _{a}^{b}f(x)g(x)dx\le \frac{f(a)+f(b)}{2}\int _{a}^{b}g(x)dx \end{aligned}$$

*holds, where*$g:\left[ a,b\right] \mathbb {\rightarrow R}$*is nonnegative, integrable and symmetric to*$(a+b)/2.$

For some results which generalize, improve and extend the inequalities (1) and (2) see Bombardelli and Varošanec (1869), İşcan (2013a, 2014c), Minculete and Mitroi (2012), Sarıkaya (2012), Tseng et al. (2011).

We recall the following inequality and special functions which are known as Beta and hypergeometric function respectively:

$$\begin{aligned} \beta \left( x,y\right)&= {} \frac{\varGamma \left( x\right) \varGamma \left( y\right) }{\varGamma \left( x+y\right) }=\int _{0}^{1}t^{x-1}\left( 1-t\right) ^{y-1}dt,\quad \text { }x,y>0,\\ _{2}F_{1}\left( a,b;c;z\right)&= {} \frac{1}{\beta \left( b,c-b\right) } \int _{0}^{1}t^{b-1}\left( 1-t\right) ^{c-b-1}\left( 1-zt\right) ^{-a}dt, \text { } \\&c> b>0,\left| z\right| <1\left( \text {see Kilbas et al. 2006}\right) . \end{aligned}$$

### **Lemma 1**

(Prudnikov et al. 1981; Wang et al. 2013) *For*$0<\alpha \le 1$*and*$0\le a<b$*we have*

$$\begin{aligned} \left| a^{\alpha }-b^{\alpha }\right| \le \left( b-a\right) ^{\alpha }. \end{aligned}$$

The following definitions and mathematical preliminaries of fractional calculus theory are used further in this paper.

### **Definition 1**

(Kilbas et al. 2006) Let $f\in L\left[ a,b\right]$. The Riemann–Liouville integrals $J_{a+}^{\alpha }f$ and $J_{b-}^{\alpha }f$ of oder $\alpha >0$ with $a\ge 0$ are defined by

$$J_{a+}^{\alpha }f(x)=\frac{1}{\varGamma (\alpha )}\int _{a}^{x}\left( x-t\right) ^{\alpha -1}f(t)dt,\quad x>a$$

and

$$J_{b-}^{\alpha }f(x)=\frac{1}{\varGamma (\alpha )}\int _{x}^{b}\left( t-x\right) ^{\alpha -1}f(t)dt,\quad x<b$$

respectively, where $$\varGamma (\alpha )$$ is the Gamma function defined by $$\varGamma (\alpha )=$$$$\int \limits _{0}^{\infty }e^{-t}t^{\alpha -1}dt$$ and $$J_{a+}^{0}f(x)=J_{b-}^{0}f(x)=f(x).$$

Because of the wide application of Hermite–Hadamard type inequalities and fractional integrals, many researchers extend their studies to Hermite–Hadamard type inequalities involving fractional integrals not limited to integer integrals. Recently, more and more Hermite–Hadamard inequalities involving fractional integrals have been obtained for different classes of functions; see Dahmani (2010), İşcan (2013b, 2014a), İşcan and Wu (2014), Mihai and Ion (2014), Sarıkaya et al. (2013), Wang et al. (2012), Wang et al. (2013).

 İşcan (2014b) can defined the so-called harmonically convex functions and established following Hermite–Hadamard type inequality for them as follows:

### **Definition 2**

Let $I\subset \mathbb {R} \backslash \left\{ 0\right\}$ be a real interval. A function $f:I\rightarrow \mathbb {R}$ is said to be harmonically convex, if

3 $$f\left( \frac{xy}{tx+\left( 1-t\right) y}\right) \le tf\left( y\right) +\left( 1-t\right) f\left( x\right)$$

for all $x,y\in I$ and $t\in \left[ 0,1\right]$. If the inequality in (3) is reversed, then *f* is said to be harmonically concave.

### **Theorem 2**

(İşcan 2014b) *Let*$f:I\subset \mathbb {R} \backslash \left\{ 0\right\} \rightarrow \mathbb {R}$*be a harmonically convex function and*$a,b\in I$. *If*$f\in L\left[ a,b \right]$*then the following inequalities holds:*

4 $$f\left( \frac{2ab}{a+b}\right) \le \frac{ab}{b-a}\int _{a}^{b}\frac{f\left( x\right) }{x^{2}}dx\le \frac{f\left( a\right) +f\left( b\right) }{2}.$$

Latif et al. (2015) gave the following definition:

### **Definition 3**

A function $g:\left[ a,b\right] \subseteq \mathbb {R} \backslash \left\{ 0\right\} \rightarrow \mathbb {R}$ is said to be harmonically symmetric with respect to $2ab/a+b$ if

$$g\left( x\right) =g\left( \frac{1}{\frac{1}{a}+\frac{1}{b}-\frac{1}{x}} \right)$$

holds for all $x\in \left[ a,b\right]$.

 Chen and Wu (2014) presented a Hermite–Hadamard–Fejer type inequality for harmonically convex functions as follows:

### **Theorem 3**

*Let*$f:I\subseteq \mathbb {R} \backslash \left\{ 0\right\} \rightarrow \mathbb {R}$*be a harmonically convex function and*$a,b\in I$. *If*$f\in L\left[ a,b \right]$ and $g:\left[ a,b\right] \subseteq \mathbb {R} \backslash \left\{ 0\right\} \rightarrow \mathbb {R}$*is nonnegative, integrable and harmonically symmetric with respect to*$\frac{2ab}{a+b}$, *then*

5 $$\begin{aligned} f\left( \frac{2ab}{a+b}\right) \int _{a}^{b}\frac{g\left( x\right) }{x^{2}}dx&\le \int _{a}^{b}\frac{f\left( x\right) g\left( x\right) }{x^{2}}dx \\&\le \frac{f\left( a\right) +f\left( b\right) }{2}\int _{a}^{b}\frac{ g\left( x\right) }{x^{2}}dx. \end{aligned}$$

In this paper, we give new Hermite–Hadamard type inequalities for harmonically convex functions in fractional integral forms. We establish new Hermite–Hadamard–Fejer inequalities for harmonically convex functions in fractional integral forms. We obtain an integral identity and some Hermite–Hadamard–Fejer type integral inequalities for harmonically convex functions in fractional integral forms.

## Main results

Throughout this section, we write $\left\| g\right\| _{\infty }= \underset{t\in \left[ a,b\right] }{\sup }\left| g(t)\right|$, for the continuous function $g:\left[ a,b\right] \mathbb {\rightarrow \mathbb {R} }$.

### **Lemma 2**

*If*$g:\left[ a,b\right] \subseteq \mathbb {R{\setminus } }\left\{ 0\right\} \mathbb {\rightarrow R}$*is integrable and harmonically symmetric with respect to*$\frac{2ab}{a+b}$, *then*

$$J_{\frac{a+b}{2ab}+}^{\alpha }\left( g\circ h\right) (1/a)=J_{\frac{a+b}{2ab} -}^{\alpha }\left( g\circ h\right) (1/b)=\frac{1}{2}\left[ \begin{array}{c} J_{\frac{a+b}{2ab}+}^{\alpha }\left( g\circ h\right) (1/a) \\ +J_{\frac{a+b}{2ab}-}^{\alpha }\left( g\circ h\right) (1/b) \end{array} \right]$$

*with*$$\alpha >0$$*and*$$h(x)=1/x$$, $$x\in \left[ \frac{1}{b},\frac{1}{a}\right]$$.

### *Proof*

Since *g* is harmonically symmetric with respect to $\frac{2ab}{a+b}$, using Definition 3 we have $g\left( \frac{1}{x}\right) =g\left(\frac{1}{(\frac{1 }{a})+(\frac{1}{b})-x}\right)$, for all $x\in \left[ \frac{1}{b},\frac{1}{a}\right]$. Hence, in the following integral setting $t=\left(\frac{1}{a})+(\frac{1}{b}\right)-x$ and $dt=-dx$ gives

$$\begin{aligned} J_{\frac{a+b}{2ab}+}^{\alpha }\left( g\circ h\right) (1/a)&= {} \frac{1}{ \varGamma (\alpha )}\int \nolimits _{\frac{a+b}{2ab}}^{\frac{1}{a}}\left( \frac{1 }{a}-t\right) ^{\alpha -1}g\left( \frac{1}{t}\right) dt \\&= {} \frac{1}{\varGamma (\alpha )}\int \nolimits _{\frac{1}{b}}^{\frac{a+b}{2ab} }\left( x-\frac{1}{b}\right) ^{\alpha -1}g\left( \frac{1}{(1/a)+(1/b)-x} \right) dx \\&= {} \frac{1}{\varGamma (\alpha )}\int \nolimits _{\frac{1}{b}}^{\frac{a+b}{2ab} }\left( x-\frac{1}{b}\right) ^{\alpha -1}g\left( \frac{1}{x}\right) dx=J_{ \frac{a+b}{2ab}-}^{\alpha }\left( g\circ h\right) (1/b). \end{aligned}$$

This completes the proof.

### **Theorem 4**

*Let*$f:I\subseteq \left( 0,\infty \right) \rightarrow \mathbb {R}$*be a function such that*$f\in L\left[ a,b\right]$, *where*$a,b\in I$. *Iffis a harmonically convex function on*$\left[ a,b\right]$, *then the following inequalities for fractional integrals holds:*

6 $$\begin{aligned} f\left( \frac{2ab}{a+b}\right) &\le \frac{\varGamma \left( \alpha +1\right) }{ 2^{1-\alpha }}\left( \frac{ab}{b-a}\right) ^{\alpha }\left\{ \begin{array}{c} J_{\frac{a+b}{2ab}+}^{\alpha }\left( f\circ h\right) \left( 1/a\right) \\ +J_{\frac{a+b}{2ab}-}^{\alpha }\left( f\circ h\right) \left( 1/b\right) \end{array} \right\} \\&\le \frac{f\left( a\right) +f\left( b\right) }{2} \end{aligned}$$

*with*$$\alpha >0$$*and*$$h(x)=1/x$$, $$x\in \left[ \frac{1}{b},\frac{1}{a}\right]$$.

### *Proof*

Since *f* is a harmonically convex function on $\left[ a,b\right]$, we have for all $t\in \left[ 0,1\right]$

7 $$\begin{aligned} f\left( \frac{2ab}{a+b}\right)&= {} f\left( \frac{2\left( \frac{ab}{ta+(1-t)b} \right) \left( \frac{ab}{tb+(1-t)a}\right) }{\left( \frac{ab}{ta+(1-t)b} \right) +\left( \frac{ab}{tb+(1-t)a}\right) }\right) \\&\le \frac{f\left( \frac{ab}{ta+(1-t)b}\right) +f\left( \frac{ab}{tb+(1-t)a }\right) }{2}. \end{aligned}$$

Multiplying both sides of (7) by $2t^{\alpha -1}$, then integrating the resulting inequality with respect to *t* over $\left[ 0, \frac{1}{2}\right]$, we obtain

$$\begin{aligned}&2f\left( \frac{2ab}{a+b}\right) \int _{0}^{\frac{1}{2}}t^{\alpha -1}dt \\&\quad \le \int _{0}^{\frac{1}{2}}t^{\alpha -1}\left[ f\left( \frac{ab}{ta+(1-t)b} \right) +f\left( \frac{ab}{tb+(1-t)a}\right) \right] dt \\&\quad =\int _{0}^{\frac{1}{2}}t^{\alpha -1}f\left( \frac{ab}{ta+(1-t)b}\right) dt+\int _{0}^{\frac{1}{2}}t^{\alpha -1}f\left( \frac{ab}{tb+(1-t)a}\right) dt. \end{aligned}$$

Setting $x=\frac{tb+(1-t)a}{ab}$ and $dx=\left( \frac{b-a}{ab}\right) dt$ gives

$$\begin{aligned} \frac{2^{1-\alpha }}{\alpha }f\left( \frac{2ab}{a+b}\right)&\le \left( \frac{ab}{b-a}\right) ^{\alpha }\left\{ \begin{array}{c} \int _{\frac{1}{b}}^{\frac{a+b}{2ab}}\left( x-\frac{1}{b}\right) ^{\alpha -1}f\left( \frac{1}{\frac{1}{a}+\frac{1}{b}-x}\right) dx \\ +\int _{\frac{1}{b}}^{\frac{a+b}{2ab}}\left( x-\frac{1}{b}\right) ^{\alpha -1}f\left( \frac{1}{x}\right) dx \end{array} \right\} \\&= {} \left( \frac{ab}{b-a}\right) ^{\alpha }\left\{ \begin{array}{c} \int _{\frac{a+b}{2ab}}^{\frac{1}{a}}\left( \frac{1}{a}-x\right) ^{\alpha -1}f\left( \frac{1}{x}\right) dx \\ +\int _{\frac{1}{b}}^{\frac{a+b}{2ab}}\left( x-\frac{1}{b}\right) ^{\alpha -1}f\left( \frac{1}{x}\right) dx \end{array} \right\} \\&= {} \left( \frac{ab}{b-a}\right) ^{\alpha }\varGamma (\alpha )\left[ J_{\frac{a+b }{2ab}+}^{\alpha }\left( f\circ h\right) (1/a)+J_{\frac{a+b}{2ab}-}^{\alpha }\left( f\circ h\right) (1/b)\right] \end{aligned}$$

and the first inequality is proved.

For the proof of the second inequality in (6), we first note that, if *f* is a harmonically convex function, then, for all $t\in \left[ 0,1\right]$, it yields

8 $$f\left( \frac{ab}{ta+(1-t)b}\right) +f\left( \frac{ab}{tb+(1-t)a}\right) \le f(a)+f(b).$$

Then multiplying both sides of (8) by $t^{\alpha -1}$ and integrating the resulting inequality with respect to *t* over $\left[ 0, \frac{1}{2}\right]$, we obtain

$$\begin{aligned}&\int _{0}^{\frac{1}{2}}t^{\alpha -1}f\left( \frac{ab}{ta+(1-t)b}\right) dt+\int _{0}^{\frac{1}{2}}t^{\alpha -1}f\left( \frac{ab}{tb+(1-t)a}\right) dt \\&\quad \le \left[ f(a)+f(b)\right] \int _{0}^{\frac{1}{2}}t^{\alpha -1}dt=\frac{ 2^{1-\alpha }}{\alpha }\frac{f(a)+f(b)}{2} \end{aligned}$$

i.e.

$$\begin{aligned}&\left( \frac{ab}{b-a}\right) ^{\alpha }\varGamma (\alpha )\left[ J_{\frac{a+b }{2ab}+}^{\alpha }\left( f\circ h\right) (1/a)+J_{\frac{a+b}{2ab}-}^{\alpha }\left( f\circ h\right) (1/b)\right] \\&\quad \le \frac{2^{1-\alpha }}{\alpha }\left( \frac{f(a)+f(b)}{2}\right) . \end{aligned}$$

The proof is completed.

### **Theorem 5**

*Let*$f:\left[ a,b\right] \mathbb {\rightarrow R}$*be a harmonicallyconvex function with*$a<b$*and*$f\in L\left[ a,b\right]$. *If*$g:\left[ a,b\right] \mathbb {\rightarrow R}$*is nonnegative, integrable and harmonically symmetric with respect to*$\frac{2ab}{a+b}$, *then the following inequalities for fractional integrals holds:*

9 $$\begin{aligned}&f\left( \frac{2ab}{a+b}\right) \left[ J_{\frac{a+b}{2ab}+}^{\alpha }\left( g\circ h\right) (1/a)+J_{\frac{a+b}{2ab}-}^{\alpha }\left( g\circ h\right) (1/b)\right] \\&\quad \le \left[ J_{\frac{a+b}{2ab}+}^{\alpha }\left( fg\circ h\right) (1/a)+J_{ \frac{a+b}{2ab}-}^{\alpha }\left( fg\circ h\right) (1/b)\right] \\&\quad \le \frac{f(a)+f(b)}{2}\left[ J_{\frac{a+b}{2ab}+}^{\alpha }\left( g\circ h\right) (1/a)+J_{\frac{a+b}{2ab}-}^{\alpha }\left( g\circ h\right) (1/b) \right] \end{aligned}$$

*with*$$\alpha >0$$*and*$$h(x)=1/x$$, $$x\in \left[ \frac{1}{b},\frac{1}{a}\right]$$.

### *Proof*

Since *f* is a harmonically convex function on $\left[ a,b\right]$, multiplying both sides of (7) by $2t^{\alpha -1}g\left( \frac{ab}{tb+(1-t)a}\right)$, then integrating the resulting inequality with respect to *t* over $\left[ 0,\frac{1}{2}\right]$, we obtain

$$\begin{aligned}&2f\left( \frac{2ab}{a+b}\right) \int _{0}^{\frac{1}{2}}t^{\alpha -1}g\left( \frac{ab}{tb+(1-t)a}\right) dt \\&\quad \le \int _{0}^{\frac{1}{2}}t^{\alpha -1}\left[ f\left( \frac{ab}{ta+(1-t)b} \right) +f\left( \frac{ab}{tb+(1-t)a}\right) \right] g\left( \frac{ab}{ tb+(1-t)a}\right) dt \\&\quad =\int _{0}^{\frac{1}{2}}t^{\alpha -1}f\left( \frac{ab}{ta+(1-t)b}\right) g\left( \frac{ab}{tb+(1-t)a}\right) dt \\&\quad \quad +\int _{0}^{\frac{1}{2}}t^{\alpha -1}f\left( \frac{ab}{tb+(1-t)a}\right) g\left( \frac{ab}{tb+(1-t)a}\right) dt. \end{aligned}$$

Since *g* is harmonically symmetric with respect to $\frac{2ab}{a+b}$, using Definition 3 we have $g\left( \frac{1}{x}\right) =g\left(\frac{1}{(\frac{1 }{a})+(\frac{1}{b})-x}\right)$, for all $x\in \left[ \frac{1}{b},\frac{1}{a}\right]$. Setting $x=\frac{tb+(1-t)a}{ab}$ and $dx=\left( \frac{b-a}{ab}\right) dt$ gives

$$\begin{aligned}&2\left( \frac{ab}{b-a}\right) ^{\alpha }f\left( \frac{2ab}{a+b}\right) \int _{\frac{1}{b}}^{\frac{a+b}{2ab}}\left( x-\frac{1}{b}\right) ^{\alpha -1}g\left( \frac{1}{x}\right) dx \\&\quad \le \left( \frac{ab}{b-a}\right) ^{\alpha }\left\{ \begin{array}{c} \int _{\frac{1}{b}}^{\frac{a+b}{2ab}}\left( x-\frac{1}{b}\right) ^{\alpha -1}f\left( \frac{1}{\frac{1}{a}+\frac{1}{b}-x}\right) g\left( \frac{1}{x} \right) dx \\ +\int _{\frac{1}{b}}^{\frac{a+b}{2ab}}\left( x-\frac{1}{b}\right) ^{\alpha -1}f\left( \frac{1}{x}\right) g\left( \frac{1}{x}\right) dx \end{array} \right\} \\&\quad =\left( \frac{ab}{b-a}\right) ^{\alpha }\left\{ \begin{array}{c} \int _{\frac{a+b}{2ab}}^{\frac{1}{a}}\left( \frac{1}{a}-x\right) ^{\alpha -1}f\left( \frac{1}{x}\right) g\left( \frac{1}{\frac{1}{a}+\frac{1}{b}-x} \right) dx \\ +\int _{\frac{1}{b}}^{\frac{a+b}{2ab}}\left( x-\frac{1}{b}\right) ^{\alpha -1}f\left( \frac{1}{x}\right) g\left( \frac{1}{x}\right) dx \end{array} \right\} \\&\quad =\left( \frac{ab}{b-a}\right) ^{\alpha }\left\{ \begin{array}{c} \int _{\frac{a+b}{2ab}}^{\frac{1}{a}}\left( \frac{1}{a}-x\right) ^{\alpha -1}f\left( \frac{1}{x}\right) g\left( \frac{1}{x}\right) dx \\ +\int _{\frac{1}{b}}^{\frac{a+b}{2ab}}\left( x-\frac{1}{b}\right) ^{\alpha -1}f\left( \frac{1}{x}\right) g\left( \frac{1}{x}\right) dx \end{array} \right\} . \end{aligned}$$

Therefore, by Lemma 2 we have

$$\begin{aligned}&\left( \frac{ab}{b-a}\right) ^{\alpha }\varGamma (\alpha )f\left( \frac{2ab}{ a+b}\right) \left[ J_{\frac{a+b}{2ab}+}^{\alpha }\left( g\circ h\right) (1/a)+J_{\frac{a+b}{2ab}-}^{\alpha }\left( g\circ h\right) (1/b)\right] \\&\quad \le \left( \frac{ab}{b-a}\right) ^{\alpha }\varGamma (\alpha )\left[ J_{ \frac{a+b}{2ab}+}^{\alpha }\left( fg\circ h\right) (1/a)+J_{\frac{a+b}{2ab} -}^{\alpha }\left( fg\circ h\right) (1/b)\right] \end{aligned}$$

and the first inequality is proved.

For the proof of the second inequality in (9) we first note that if *f* is a harmonically convex function, then, multiplying both sides of (8) by $t^{\alpha -1}g\left( \frac{ab}{ tb+(1-t)a}\right)$ and integrating the resulting inequality with respect to *t* over $\left[ 0,\frac{1}{2}\right]$, we obtain

$$\begin{aligned}&\int _{0}^{\frac{1}{2}}t^{\alpha -1}f\left( \frac{ab}{ta+(1-t)b}\right) g\left( \frac{ab}{tb+(1-t)a}\right) dt \\&\quad\quad +\int _{0}^{\frac{1}{2}}t^{\alpha -1}f\left( \frac{ab}{tb+(1-t)a}\right) g\left( \frac{ab}{tb+(1-t)a}\right) dt \\&\quad \le \left[ f(a)+f(b)\right] \int _{0}^{\frac{1}{2}}t^{\alpha -1}g\left( \frac{ab}{tb+(1-t)a}\right) dt \end{aligned}$$

i.e.

$$\begin{aligned}&\left( \frac{ab}{b-a}\right) ^{\alpha }\varGamma (\alpha )\left[ J_{\frac{a+b }{2ab}+}^{\alpha }\left( fg\circ h\right) (1/a)+J_{\frac{a+b}{2ab}-}^{\alpha }\left( fg\circ h\right) (1/b)\right] \\&\quad \le \left( \frac{ab}{b-a}\right) ^{\alpha }\varGamma (\alpha )\left( \frac{ f(a)+f(b)}{2}\right) \left[ J_{\frac{a+b}{2ab}+}^{\alpha }\left( g\circ h\right) (1/a)+J_{\frac{a+b}{2ab}-}^{\alpha }\left( g\circ h\right) (1/b) \right] . \end{aligned}$$

The proof is completed.

### *Remark 1*

In Theorem 5,

(i) if we take $\alpha =1$, then inequality (9) becomes inequality (5) of Theorem 3.
(ii) if we take $g(x)=1$, then inequality (9) becomes inequality (6) of Theorem 4.
(iii) if we take $\alpha =1$ and $g(x)=1$, then inequality (9) becomes inequality (4) of Theorem 2.

### **Lemma 3**

*Let*$f:I\subset \left( 0,\infty \right) \rightarrow \mathbb {R}$*be a differentiable function on*$I {{}^\circ }$*, the interior of I, such that *$f^{\prime }\in L\left[ a,b\right]$*, where*$a,b\in I$*. If *$g:\left[ a,b\right] \mathbb {\rightarrow R}$*is integrable and harmonically symmetric with respect to*$\frac{2ab}{a+b}$*, then the following equality for fractional integrals holds:*

10 $$\begin{aligned}&f\left( \frac{2ab}{a+b}\right) \left[ J_{\frac{a+b}{2ab}+}^{\alpha }\left( g\circ h\right) (1/a)+J_{\frac{a+b}{2ab}-}^{\alpha }\left( g\circ h\right) (1/b)\right] \\&\quad \quad -\left[ J_{\frac{a+b}{2ab}+}^{\alpha }\left( fg\circ h\right) (1/a)+J_{ \frac{a+b}{2ab}-}^{\alpha }\left( fg\circ h\right) (1/b)\right] \\&\quad =\frac{1}{\varGamma (\alpha )}\left[ \begin{array}{c} \int _{\frac{1}{b}}^{\frac{a+b}{2ab}}\left( \int _{\frac{1}{b}}^{t}\left( s- \frac{1}{b}\right) ^{\alpha -1}\left( g\circ h\right) (s)ds\right) \left( f\circ h\right) ^{\prime }(t)dt \\ -\int _{\frac{a+b}{2ab}}^{\frac{1}{a}}\left( \int _{t}^{\frac{1}{a}}\left( \frac{1}{a}-s\right) ^{\alpha -1}\left( g\circ h\right) (s)ds\right) \left( f\circ h\right) ^{\prime }(t)dt \end{array} \right] \end{aligned}$$

*with *$$\alpha >0$$*and *$$h(x)=1/x$$, $$x\in \left[ \frac{1}{b},\frac{1}{a}\right]$$.

### *Proof*

It suffices to note that

$$\begin{aligned} I&= {} \int _{\frac{1}{b}}^{\frac{a+b}{2ab}}\left( \int _{\frac{1}{b}}^{t}\left( s-\frac{1}{b}\right) ^{\alpha -1}\left( g\circ h\right) (s)ds\right) \left( f\circ h\right) ^{\prime }(t)dt \\&\quad-\int _{\frac{a+b}{2ab}}^{\frac{1}{a}}\left( \int _{t}^{\frac{1}{a}}\left( \frac{1}{a}-s\right) ^{\alpha -1}\left( g\circ h\right) (s)ds\right) \left( f\circ h\right) ^{\prime }(t)dt \\&= {} I_{1}-I_{2}. \end{aligned}$$

By integration by parts and Lemma 2 we get

$$\begin{aligned} I_{1}&= {} \left. \left( \int _{\frac{1}{b}}^{t}\left( s-\frac{1}{b}\right) ^{\alpha -1}\left( g\circ h\right) (s)ds\right) \left( f\circ h\right) (t)\right| _{\frac{1}{b}}^{\frac{a+b}{2ab}} \\&\quad-\int _{\frac{1}{b}}^{\frac{a+b}{2ab}}\left( t-\frac{1}{b}\right) ^{\alpha -1}\left( g\circ h\right) (t)\left( f\circ h\right) (t)dt \\&= {} \left( \int _{\frac{1}{b}}^{\frac{a+b}{2ab}}\left( s-\frac{1}{b}\right) ^{\alpha -1}\left( g\circ h\right) (s)ds\right) f\left(\frac{2ab}{a+b}\right) \\&\quad-\int _{\frac{1}{b}}^{\frac{a+b}{2ab}}\left( t-\frac{1}{b}\right) ^{\alpha -1}\left( g\circ h\right) (t)\left( f\circ h\right) (t)dt \\&= {} \varGamma (\alpha )\left[ f\left(\frac{2ab}{a+b}\right)J_{\frac{a+b}{2ab}-}^{\alpha }\left( g\circ h\right) (1/b)-J_{\frac{a+b}{2ab}-}^{\alpha }\left( fg\circ h\right) (1/b)\right] \\&= {} \varGamma (\alpha )\left[ \begin{array}{l} \frac{f\left(\frac{2ab}{a+b}\right)}{2}\left[ J_{\frac{a+b}{2ab}+}^{\alpha }\left( g\circ h\right) (1/a)+J_{\frac{a+b}{2ab}-}^{\alpha }\left( g\circ h\right) (1/b)\right] \\ -J_{\frac{a+b}{2ab}-}^{\alpha }\left( fg\circ h\right) (1/b) \end{array} \right] \end{aligned}$$

and similarly

$$\begin{aligned} I_{2}&= {} \left. \left( \int _{t}^{\frac{1}{a}}\left( \frac{1}{a}-s\right) ^{\alpha -1}\left( g\circ h\right) (s)ds\right) \left( f\circ h\right) (t)\right| _{\frac{a+b}{2ab}}^{\frac{1}{a}} \\&\quad+\int _{\frac{a+b}{2ab}}^{\frac{1}{a}}\left( \frac{1}{a}-t\right) ^{\alpha -1}\left( g\circ h\right) (t)\left( f\circ h\right) (t)dt \\&= {} -\left( \int _{\frac{a+b}{2ab}}^{\frac{1}{a}}\left( \frac{1}{a}-s\right) ^{\alpha -1}\left( g\circ h\right) (s)ds\right) f\left( \frac{2ab}{a+b}\right) \\&\quad+\int _{\frac{a+b}{2ab}}^{\frac{1}{a}}\left( \frac{1}{a}-t\right) ^{\alpha -1}\left( g\circ h\right) (t)\left( f\circ h\right) (t)dt \\&= {} \varGamma (\alpha )\left[ -f(\frac{2ab}{a+b})J_{\frac{a+b}{2ab}+}^{\alpha }\left( g\circ h\right) (1/a)+J_{\frac{a+b}{2ab}+}^{\alpha }\left( fg\circ h\right) (1/a)\right] \\&= {} \varGamma (\alpha )\left[ \begin{array}{l} -\frac{f(\frac{2ab}{a+b})}{2}\left[ J_{\frac{a+b}{2ab}+}^{\alpha }\left( g\circ h\right) (1/a)+J_{\frac{a+b}{2ab}-}^{\alpha }\left( g\circ h\right) (1/b)\right] \\ +J_{\frac{a+b}{2ab}+}^{\alpha }\left( fg\circ h\right) (1/a) \end{array} \right] . \end{aligned}$$

Thus, we can write

$$\begin{aligned} I=I_{1}-I_{2}=\varGamma (\alpha )\left\{ \begin{array}{l} f\left( \frac{2ab}{a+b}\right) \left[ J_{\frac{a+b}{2ab}+}^{\alpha }\left( g\circ h\right) (1/a)+J_{\frac{a+b}{2ab}-}^{\alpha }\left( g\circ h\right) (1/b)\right] \\ -\left[ J_{\frac{a+b}{2ab}+}^{\alpha }\left( fg\circ h\right) (1/a)+J_{\frac{ a+b}{2ab}-}^{\alpha }\left( fg\circ h\right) (1/b)\right] \end{array} \right\} . \end{aligned}$$

Multiplying both sides by $\left( \varGamma (\alpha )\right) ^{-1}$ we obtain (10). This completes the proof.

### **Theorem 6**

*Let*$f:I\subset \left( 0,\infty \right) \rightarrow \mathbb {R}$*be a differentiable function on*$I {{}^\circ }$, *the interior ofI*, *such that*$f^{\prime }\in L\left[ a,b\right]$, *where*$a,b\in I$*and*$a<b$. *If*$\left| f^{\prime }\right|$*is harmonically convex on*$\left[ a,b\right]$, $g:\left[ a,b\right] \mathbb { \rightarrow R}$*is continuous and harmonically symmetric with respect to*$\frac{2ab}{a+b}$, *then the following inequality for fractional integrals holds:*

11 $$\begin{aligned}&\left| \begin{array}{c} f\left( \frac{2ab}{a+b}\right) \left[ J_{\frac{a+b}{2ab}+}^{\alpha }\left( g\circ h\right) (1/a)+J_{\frac{a+b}{2ab}-}^{\alpha }\left( g\circ h\right) (1/b)\right] \\ -\left[ J_{\frac{a+b}{2ab}+}^{\alpha }\left( fg\circ h\right) (1/a)+J_{\frac{ a+b}{2ab}-}^{\alpha }\left( fg\circ h\right) (1/b)\right] \end{array} \right| \\&\quad \le \frac{\left\| g\right\| _{\infty }ab\left( b-a\right) }{\varGamma (\alpha +1)}\left( \frac{b-a}{ab}\right) ^{\alpha }\left[ C_{1}\left( \alpha \right) \left| f^{\prime }\left( a\right) \right| +C_{2}\left( \alpha \right) \left| f^{\prime }\left( b\right) \right| \right] \end{aligned}$$

*where*

$$\begin{aligned} C_{1}\left( \alpha \right)&= {} \left[ \begin{array}{c} \frac{b^{-2}}{\left( \alpha +1\right) \left( \alpha +2\right) } \begin{array}{c} _{2}F_{1}\left( 2,\alpha +1;\alpha +3;1-\frac{a}{b}\right) \end{array} \\ -\frac{\left( a+b\right) ^{-2}}{\left( \alpha +1\right) \left( \alpha +2\right) } \begin{array}{c} _{2}F_{1}\left( 2,\alpha +1;\alpha +3;\frac{b-a}{b+a}\right) \end{array} \end{array} \right] ,\\ C_{2}\left( \alpha \right)&= {} \left[ \begin{array}{c} \frac{b^{-2}}{\alpha +2} \begin{array}{c} _{2}F_{1}\left( 2,\alpha +2;\alpha +3;1-\frac{a}{b}\right) \end{array} \\ -\frac{2\left( a+b\right) ^{-2}}{\alpha +1} \begin{array}{c} _{2}F_{1}\left( 2,\alpha +1;\alpha +2;\frac{b-a}{b+a}\right) \end{array} \\ +\frac{\left( a+b\right) ^{-2}}{\left( \alpha +1\right) \left( \alpha +2\right) } \begin{array}{c} _{2}F_{1}\left( 2,\alpha +1;\alpha +3;\frac{b-a}{b+a}\right) \end{array} \end{array} \right] , \end{aligned}$$

*with*$$0<\alpha \le 1$$*and*$$h(x)=1/x$$, $$x\in \left[ \frac{1}{b},\frac{1}{a} \right]$$.

### *Proof*

From Lemma 3 we have

$$\begin{aligned}&\left| \begin{array}{c} f\left( \frac{2ab}{a+b}\right) \left[ J_{\frac{a+b}{2ab}+}^{\alpha }\left( g\circ h\right) (1/a)+J_{\frac{a+b}{2ab}-}^{\alpha }\left( g\circ h\right) (1/b)\right] \\ -\left[ J_{\frac{a+b}{2ab}+}^{\alpha }\left( fg\circ h\right) (1/a)+J_{\frac{ a+b}{2ab}-}^{\alpha }\left( fg\circ h\right) (1/b)\right] \end{array} \right| \\&\quad \le \frac{1}{\varGamma (\alpha )}\left[ \begin{array}{c} \int _{\frac{1}{b}}^{\frac{a+b}{2ab}}\left( \int _{\frac{1}{b}}^{t}\left( s- \frac{1}{b}\right) ^{\alpha -1}\left| \left( g\circ h\right) (s)\right| ds\right) \left| \left( f\circ h\right) ^{\prime }(t)\right| dt \\ +\int _{\frac{a+b}{2ab}}^{\frac{1}{a}}\left( \int _{t}^{\frac{1}{a}}\left( \frac{1}{a}-s\right) ^{\alpha -1}\left| \left( g\circ h\right) (s)\right| ds\right) \left| \left( f\circ h\right) ^{\prime }(t)\right| dt \end{array} \right] \\&\quad \le \frac{\left\| g\right\| _{\infty }}{\varGamma (\alpha )}\left[ \begin{array}{c} \int _{\frac{1}{b}}^{\frac{a+b}{2ab}}\left( \int _{\frac{1}{b}}^{t}\left( s- \frac{1}{b}\right) ^{\alpha -1}ds\right) \left| \left( f\circ h\right) ^{\prime }(t)\right| dt \\ +\int _{\frac{a+b}{2ab}}^{\frac{1}{a}}\left( \int _{t}^{\frac{1}{a}}\left( \frac{1}{a}-s\right) ^{\alpha -1}ds\right) \left| \left( f\circ h\right) ^{\prime }(t)\right| dt \end{array} \right] \\&\quad =\frac{\left\| g\right\| _{\infty }}{\varGamma (\alpha )}\left[ \begin{array}{c} \int _{\frac{1}{b}}^{\frac{a+b}{2ab}}\frac{\left( t-\frac{1}{b}\right) ^{\alpha }}{\alpha }\frac{1}{t^{2}}\left| f^{\prime }(\frac{1}{t} )\right| dt \\ +\int _{\frac{a+b}{2ab}}^{\frac{1}{a}}\frac{\left( \frac{1}{a}-t\right) ^{\alpha }}{\alpha }\frac{1}{t^{2}}\left| f^{\prime }(\frac{1}{t} )\right| dt \end{array} \right] . \end{aligned}$$

Setting $t=\frac{ub+(1-u)a}{ab}$ and $dt=\left( \frac{b-a}{ab}\right) du$ gives

12 $$\begin{aligned}&\left| \begin{array}{c} f\left( \frac{2ab}{a+b}\right) \left[ J_{\frac{a+b}{2ab}+}^{\alpha }\left( g\circ h\right) (1/a)+J_{\frac{a+b}{2ab}-}^{\alpha }\left( g\circ h\right) (1/b)\right] \\ -\left[ J_{\frac{a+b}{2ab}+}^{\alpha }\left( fg\circ h\right) (1/a)+J_{\frac{ a+b}{2ab}-}^{\alpha }\left( fg\circ h\right) (1/b)\right] \end{array} \right| \\&\quad \le \frac{\left\| g\right\| _{\infty }ab\left( b-a\right) }{\varGamma (\alpha +1)}\left( \frac{b-a}{ab}\right) ^{\alpha }\left[ \begin{array}{c} \int _{0}^{\frac{1}{2}}\frac{u^{\alpha }}{\left( ub+\left( 1-u\right) a\right) ^{2}}\left| f^{\prime }(\frac{ab}{ub+\left( 1-u\right) a} )\right| du \\ +\int _{\frac{1}{2}}^{1}\frac{\left( 1-u\right) ^{\alpha }}{\left( ub+\left( 1-u\right) a\right) ^{2}}\left| f^{\prime }\left( \frac{ab}{ub+\left( 1-u\right) a}\right) \right| du \end{array} \right] . \end{aligned}$$

Since $\left| f^{\prime }\right|$ is harmonically convex on $\left[ a,b\right]$, we have

13 $$\begin{aligned} \left| f^{\prime }\left(\frac{ab}{ub+(1-u)at}\right)\right| \le u\left| f^{\prime }\left( a\right) \right| +\left( 1-u\right) \left| f^{\prime }\left( b\right) \right| . \end{aligned}$$

If we use (13) in (12) , we have

14 $$\begin{aligned}&\left| \begin{array}{c} f\left( \frac{2ab}{a+b}\right) \left[ J_{\frac{a+b}{2ab}+}^{\alpha }\left( g\circ h\right) (1/a)+J_{\frac{a+b}{2ab}-}^{\alpha }\left( g\circ h\right) (1/b)\right] \\ -\left[ J_{\frac{a+b}{2ab}+}^{\alpha }\left( fg\circ h\right) (1/a)+J_{\frac{ a+b}{2ab}-}^{\alpha }\left( fg\circ h\right) (1/b)\right] \end{array} \right| \\&\quad \le \frac{\left\| g\right\| _{\infty }ab\left( b-a\right) }{\varGamma (\alpha +1)}\left( \frac{b-a}{ab}\right) ^{\alpha } \\&\quad \quad \times \left[ \begin{array}{c} \int _{0}^{\frac{1}{2}}\frac{u^{\alpha }}{\left( ub+\left( 1-u\right) a\right) ^{2}}\left[ u\left| f^{\prime }\left( a\right) \right| +\left( 1-u\right) \left| f^{\prime }\left( b\right) \right| \right] du \\ +\int _{\frac{1}{2}}^{1}\frac{\left( 1-u\right) ^{\alpha }}{\left( ub+\left( 1-u\right) a\right) ^{2}}\left[ u\left| f^{\prime }\left( a\right) \right| +\left( 1-u\right) \left| f^{\prime }\left( b\right) \right| \right] du \end{array} \right] . \end{aligned}$$

Calculating the following integrals by Lemma 1, we have

15 $$\begin{aligned}&\int _{0}^{\frac{1}{2}}\frac{u^{\alpha +1}}{\left( ub+(1-u)a\right) ^{2}} du+\int _{\frac{1}{2}}^{1}\frac{\left( 1-u\right) ^{\alpha }u}{\left( ub+(1-u)a\right) ^{2}}du \\&\quad =\int _{0}^{1}\frac{\left( 1-u\right) ^{\alpha }u}{\left( ub+(1-u)a\right) ^{2}}du-\int _{0}^{\frac{1}{2}}\frac{\left( 1-u\right) ^{\alpha }-u^{\alpha } }{\left( ub+(1-u)a\right) ^{2}}udu \\&\quad \le \int _{0}^{1}\frac{\left( 1-u\right) ^{\alpha }u}{\left( ub+(1-u)a\right) ^{2}}du-\int _{0}^{\frac{1}{2}}\frac{\left( 1-2u\right) ^{\alpha }}{\left( ub+(1-u)a\right) ^{2}}udu \\&\quad =\int _{0}^{1}\frac{\left( 1-u\right) ^{\alpha }u}{\left( ub+(1-u)a\right) ^{2}}du-\frac{1}{4}\int _{0}^{1}\frac{\left( 1-u\right) ^{\alpha }}{\left( \frac{u}{2}b+(1-\frac{u}{2})a\right) ^{2}}udu \\&\quad =\int _{0}^{1}\left( 1-u\right) u^{\alpha }b^{-2}\left( 1-u\left( 1-\frac{a }{b}\right) \right) ^{-2}du \\&\quad \quad -\frac{1}{4}\int _{0}^{1}\left( 1-v\right) v^{\alpha }\left( \frac{a+b}{2} \right) ^{-2}\left( 1-v\left( \frac{b-a}{b+a}\right) \right) ^{-2}dv \\&\quad =\left[ \begin{array}{c} \frac{b^{-2}}{\left( \alpha +1\right) \left( \alpha +2\right) } \begin{array}{c} _{2}F_{1}\left( 2,\alpha +1;\alpha +3;1-\frac{a}{b}\right) \end{array} \\ -\frac{\left( a+b\right) ^{-2}}{\left( \alpha +1\right) \left( \alpha +2\right) } \begin{array}{c} _{2}F_{1}\left( 2,\alpha +1;\alpha +3;\frac{b-a}{b+a}\right) \end{array} \end{array} \right] \\&\quad =C_{1}\left( \alpha \right) \end{aligned}$$

and similarly we get

16 $$\begin{aligned}&\int _{0}^{\frac{1}{2}}\frac{u^{\alpha }}{\left( ub+(1-u)a\right) ^{2}} \left( 1-u\right) du+\int _{\frac{1}{2}}^{1}\frac{\left( 1-u\right) ^{\alpha } }{\left( ub+(1-u)a\right) ^{2}}\left( 1-u\right) du \\&\quad =\int _{0}^{1}\frac{\left( 1-u\right) ^{\alpha +1}}{\left( ub+(1-u)a\right) ^{2}}du-\int _{0}^{\frac{1}{2}}\frac{\left( 1-u\right) ^{\alpha }-u^{\alpha } }{\left( ub+(1-u)a\right) ^{2}}\left( 1-u\right) du \\&\quad \le \int _{0}^{1}\frac{\left( 1-u\right) ^{\alpha +1}}{\left( ub+(1-u)a\right) ^{2}}du-\int _{0}^{\frac{1}{2}}\frac{\left( 1-2u\right) ^{\alpha }}{\left( ub+(1-u)a\right) ^{2}}\left( 1-u\right) du \\&\quad =\int _{0}^{1}\frac{\left( 1-u\right) ^{\alpha +1}}{\left( ub+(1-u)a\right) ^{2}}du-\int _{0}^{\frac{1}{2}}\frac{\left( 1-2u\right) ^{\alpha }}{\left( ub+(1-u)a\right) ^{2}}du \\&\quad \quad +\int _{0}^{\frac{1}{2}}\frac{u\left( 1-2u\right) ^{\alpha }}{\left( ub+(1-u)a\right) ^{2}}du \\&\quad =\int _{0}^{1}\frac{u^{\alpha +1}}{\left( ua+(1-u)b\right) ^{2}}du-\frac{1}{ 2}\int _{0}^{1}\frac{\left( 1-u\right) ^{\alpha }}{\left( \frac{u}{2}b+(1- \frac{u}{2})a\right) ^{2}}du \\&\quad \quad +\frac{1}{4}\int _{0}^{1}\frac{u\left( 1-u\right) ^{\alpha }}{\left( \frac{u }{2}b+(1-\frac{u}{2})a\right) ^{2}}du \\&\quad =\int _{0}^{1}\frac{u^{\alpha +1}}{\left( ua+(1-u)b\right) ^{2}}du-\frac{1}{ 2}\int _{0}^{1}v^{\alpha }\left( \frac{a+b}{2}\right) ^{-2}\left( 1-v\left( \frac{b-a}{b+a}\right) \right) ^{-2}dv \\&\quad \quad +\frac{1}{4}\int _{0}^{1}\left( 1-v\right) v^{\alpha }\left( \frac{a+b}{2} \right) ^{-2}\left( 1-v\left( \frac{b-a}{b+a}\right) \right) ^{-2}dv \\&\quad =\left[ \begin{array}{c} \frac{b^{-2}}{\alpha +2} \begin{array}{c} _{2}F_{1}\left( 2,\alpha +2;\alpha +3;1-\frac{a}{b}\right) \end{array} \\ -\frac{2\left( a+b\right) ^{-2}}{\alpha +1} \begin{array}{c} _{2}F_{1}\left( 2,\alpha +1;\alpha +2;\frac{b-a}{b+a}\right) \end{array} \\ +\frac{\left( a+b\right) ^{-2}}{\left( \alpha +1\right) \left( \alpha +2\right) } \begin{array}{c} _{2}F_{1}\left( 2,\alpha +1;\alpha +3;\frac{b-a}{b+a}\right) \end{array} \end{array} \right] \\&\quad =C_{2}\left( \alpha \right) . \end{aligned}$$

If we use (15) and (16) in (14) , we have (11). This completes the proof.

### **Corollary 1**

*In Theorem 6:*

*(1) If we take*$\alpha =1$*we have the following Hermite–Hadamard–Fejer inequality for harmonically convex functions which is related to the left-hand side of (*5*):*

$$\begin{aligned}&\left| f\left( \frac{2ab}{a+b}\right) \int _{a}^{b}\frac{g\left( x\right) }{x^{2}}dx-\int _{a}^{b}\frac{f\left( x\right) g\left( x\right) }{ x^{2}}dx\right| \\&\quad \le \left\| g\right\| _{\infty }\left( b-a\right) ^{2}\left[ C_{1}\left( 1\right) \left| f^{\prime }\left( a\right) \right| +C_{2}\left( 1\right) \left| f^{\prime }\left( b\right) \right| \right] , \end{aligned}$$

*(2) If we take*$g\left( x\right) =1$*we have following Hermite–Hadamard type inequality for harmonically convex functions in fractional integral forms which is related to the left-hand side of (*6*):*

$$\begin{aligned}&\left| f\left( \frac{2ab}{a+b}\right) -\frac{\varGamma \left( \alpha +1\right) }{2^{1-\alpha }}\left( \frac{ab}{b-a}\right) ^{\alpha }\left\{ \begin{array}{c} J_{\frac{a+b}{2ab}+}^{\alpha }\left( f\circ h\right) \left( 1/a\right) \\ +J_{\frac{a+b}{2ab}-}^{\alpha }\left( f\circ h\right) \left( 1/b\right) \end{array} \right\} \right| \\&\quad \le \frac{ab\left( b-a\right) }{2^{1-\alpha }}\left[ C_{1}\left( \alpha \right) \left| f^{\prime }\left( a\right) \right| +C_{2}\left( \alpha \right) \left| f^{\prime }\left( b\right) \right| \right] , \end{aligned}$$

*(3) If we take*$\alpha =1$*and*$g\left( x\right) =1$*we have the following Hermite–Hadamard type inequality for harmonically convex functions which is related to the left-hand side of (*4*):*

$$\left| f\left( \frac{2ab}{a+b}\right) -\frac{ab}{b-a}\int _{a}^{b}\frac{ f\left( x\right) }{x^{2}}dx\right| \le ab\left( b-a\right) \left[ C_{1}\left( 1\right) \left| f^{\prime }\left( a\right) \right| +C_{2}\left( 1\right) \left| f^{\prime }\left( b\right) \right| \right] .$$

### **Theorem 7**

*Let*$f:I\subset \left( 0,\infty \right) \rightarrow \mathbb {R}$*be a differentiable function on*$I {{}^\circ }$, *the interior ofI*, *such that*$f^{\prime }\in L\left[ a,b\right]$, *where*$a,b\in I$. *If*$\left| f^{\prime }\right| ^{q},q\ge 1,$*is harmonicallyconvex on*$\left[ a,b\right]$, $g:\left[ a,b\right] \mathbb { \rightarrow R}$*is continuous and harmonically symmetric with respect to*$\frac{2ab}{a+b}$, *then the following inequality for fractional integrals holds:*

17 $$\begin{aligned}&\left| \begin{array}{c} \frac{f(a)+f(b)}{2}\left[ J_{1/b+}^{\alpha }\left( g\circ h\right) (1/a)+J_{1/a-}^{\alpha }\left( g\circ h\right) (1/b)\right] \\ -\left[ J_{1/b+}^{\alpha }\left( fg\circ h\right) (1/a)+J_{1/a-}^{\alpha }\left( fg\circ h\right) (1/b)\right] \end{array} \right| \\&\quad \le \frac{\left\| g\right\| _{\infty }ab\left( b-a\right) }{\varGamma (\alpha +1)}\left( \frac{b-a}{ab}\right) ^{\alpha } \\&\quad \quad \times \left[ \begin{array}{l} C_{3}^{^{1-\frac{1}{q}}}\left( \alpha \right) \left[ \left( \begin{array}{c} C_{4}\left( \alpha \right) \left| f^{\prime }(a)\right| ^{q} \\ +C_{5}\left( \alpha \right) \left| f^{\prime }(b)\right| ^{q} \end{array} \right) \right] ^{\frac{1}{q}} \\ +C_{6}^{1-\frac{1}{q}}\left( \alpha \right) \left[ \left( \begin{array}{c} C_{7}\left( \alpha \right) \left| f^{\prime }(a)\right| ^{q} \\ +C_{8}\left( \alpha \right) \left| f^{\prime }(b)\right| ^{q} \end{array} \right) \right] ^{\frac{1}{q}} \end{array} \right] \end{aligned}$$

*where*

$$\begin{aligned} C_{3}\left( \alpha \right)&= {} \frac{\left( a+b\right) ^{-2}}{2^{\alpha -1}\left( \alpha +1\right) } \begin{array}{c} _{2}F_{1}\left( 2,1;\alpha +2;\frac{b-a}{b+a}\right) \end{array} , \\ C_{4}\left( \alpha \right)&= {} \frac{\left( a+b\right) ^{-2}}{2^{\alpha }\left( \alpha +2\right) } \begin{array}{c} _{2}F_{1}\left( 2,1;\alpha +3;\frac{b-a}{b+a}\right) \end{array} , \\ C_{5}\left( \alpha \right)&= {} C_{3}\left( \alpha \right) -C_{4}\left( \alpha \right) , \\ \text { }C_{6}\left( \alpha \right)&= {} \frac{b^{-2}}{2^{\alpha +1}\left( \alpha +1\right) } \begin{array}{c} _{2}F_{1}\left( 2,\alpha +1;\alpha +2;\frac{1}{2}\left( 1-\frac{a}{b}\right) \right) \end{array} , \\ C_{7}\left( \alpha \right)&= {} \left[ \begin{array}{c} \frac{b^{-2}}{2^{\alpha +1}\left( \alpha +1\right) } \begin{array}{c} _{2}F_{1}\left( 2,\alpha +1;\alpha +2;\frac{1}{2}\left( 1-\frac{a}{b}\right) \right) \end{array} \\ -\frac{b^{-2}}{2^{\alpha +2}\left( \alpha +2\right) } \begin{array}{c} _{2}F_{1}\left( 2,\alpha +2;\alpha +3;\frac{1}{2}\left( 1-\frac{a}{b}\right) \right) \end{array} \end{array} \right] , \\ C_{8}\left( \alpha \right)&= {} C_{6}\left( \alpha \right) -C_{7}\left( \alpha \right) , \end{aligned}$$

*with*$$\alpha >1$$*and*$$h(x)=1/x$$, $$x\in \left[ \frac{1}{b},\frac{1}{a}\right]$$.

### *Proof*

Using (12) , power mean inequality and the harmonically convexity of $\left| f^{\prime }\right| ^{q}$, it follows that

18 $$\begin{aligned}&\left| \begin{array}{c} f\left( \frac{2ab}{a+b}\right) \left[ J_{\frac{a+b}{2ab}+}^{\alpha }\left( g\circ h\right) (1/a)+J_{\frac{a+b}{2ab}-}^{\alpha }\left( g\circ h\right) (1/b)\right] \\ -\left[ J_{\frac{a+b}{2ab}+}^{\alpha }\left( fg\circ h\right) (1/a)+J_{\frac{ a+b}{2ab}-}^{\alpha }\left( fg\circ h\right) (1/b)\right] \end{array} \right| \\&\quad \le \frac{\left\| g\right\| _{\infty }ab\left( b-a\right) }{\varGamma (\alpha +1)}\left( \frac{b-a}{ab}\right) ^{\alpha }\left[ \begin{array}{c} \int _{0}^{\frac{1}{2}}\frac{u^{\alpha }}{\left( ub+\left( 1-u\right) a\right) ^{2}}\left| f^{\prime }(\frac{ab}{ub+\left( 1-u\right) a} )\right| du \\ +\int _{\frac{1}{2}}^{1}\frac{\left( 1-u\right) ^{\alpha }}{\left( ub+\left( 1-u\right) a\right) ^{2}}\left| f^{\prime }\left(\frac{ab}{ub+\left( 1-u\right) a}\right)\right| du \end{array} \right] \\&\quad \le \frac{\left\| g\right\| _{\infty }ab\left( b-a\right) }{\varGamma (\alpha +1)}\left( \frac{b-a}{ab}\right) ^{\alpha } \\&\quad \left[ \begin{array}{c} \left( \int _{0}^{\frac{1}{2}}\frac{u^{\alpha }}{\left( ub+\left( 1-u\right) a\right) ^{2}}du\right) ^{1-\frac{1}{q}} \\ \times \left( \int _{0}^{\frac{1}{2}}\frac{u^{\alpha }}{\left( ub+\left( 1-u\right) a\right) ^{2}}\left| f^{\prime }\left(\frac{ab}{ub+(1-u)a} \right)\right| ^{q}du\right) ^{\frac{1}{q}} \\ +\left( \int _{\frac{1}{2}}^{1}\frac{\left( 1-u\right) ^{\alpha }}{\left( ub+\left( 1-u\right) a\right) ^{2}}du\right) ^{1-\frac{1}{q}} \\ \times \left( \int _{\frac{1}{2}}^{1}\frac{\left( 1-u\right) ^{\alpha }}{ \left( ub+\left( 1-u\right) a\right) ^{2}}\left| f^{\prime }\left(\frac{ab}{ ub+(1-u)a}\right)\right| ^{q}du\right) ^{\frac{1}{q}} \end{array} \right] \\&\quad \le \frac{\left\| g\right\| _{\infty }ab\left( b-a\right) }{\varGamma (\alpha +1)}\left( \frac{b-a}{ab}\right) ^{\alpha } \\&\quad \times \left[ \begin{array}{c} \left( \int _{0}^{\frac{1}{2}}\frac{u^{\alpha }}{\left( ub+\left( 1-u\right) a\right) ^{2}}du\right) ^{1-\frac{1}{q}} \\ \times \left( \int _{0}^{\frac{1}{2}}\frac{u^{\alpha }}{\left( ub+\left( 1-u\right) a\right) ^{2}}\left[ u\left| f^{\prime }(a)\right| ^{q}+\left( 1-u\right) \left| f^{\prime }(b)\right| ^{q}\right] du\right) ^{\frac{1}{q}} \\ +\left( \int _{\frac{1}{2}}^{1}\frac{\left( 1-u\right) ^{\alpha }}{\left( ub+\left( 1-u\right) a\right) ^{2}}du\right) ^{1-\frac{1}{q}} \\ \times \left( \int _{\frac{1}{2}}^{1}\frac{\left( 1-u\right) ^{\alpha }}{ \left( ub+\left( 1-u\right) a\right) ^{2}}\left[ u\left| f^{\prime }(a)\right| ^{q}+\left( 1-u\right) \left| f^{\prime }(b)\right| ^{q}\right] du\right) ^{\frac{1}{q}} \end{array} \right] \\&\quad =\frac{\left\| g\right\| _{\infty }ab\left( b-a\right) }{\varGamma (\alpha +1)}\left( \frac{b-a}{ab}\right) ^{\alpha } \\&\quad \times \left[ \begin{array}{c} \left( \int _{0}^{\frac{1}{2}}\frac{u^{\alpha }}{\left( ub+\left( 1-u\right) a\right) ^{2}}du\right) ^{1-\frac{1}{q}} \\ \times \left( \begin{array}{c} \int _{0}^{\frac{1}{2}}\frac{u^{\alpha +1}}{\left( ub+\left( 1-u\right) a\right) ^{2}}du\left| f^{\prime }(a)\right| ^{q} \\ +\int _{0}^{\frac{1}{2}}\frac{u^{\alpha }}{\left( ub+\left( 1-u\right) a\right) ^{2}}\left( 1-u\right) du\left| f^{\prime }(b)\right| ^{q} \end{array} \right) ^{\frac{1}{q}} \\ +\left( \int _{\frac{1}{2}}^{1}\frac{\left( 1-u\right) ^{\alpha }}{\left( ub+\left( 1-u\right) a\right) ^{2}}du\right) ^{1-\frac{1}{q}} \\ \times \left( \begin{array}{c} \int _{\frac{1}{2}}^{1}\frac{\left( 1-u\right) ^{\alpha }}{\left( ub+\left( 1-u\right) a\right) ^{2}}udu\left| f^{\prime }(a)\right| ^{q} \\ +\int _{\frac{1}{2}}^{1}\frac{\left( 1-u\right) ^{\alpha +1}}{\left( ub+\left( 1-u\right) a\right) ^{2}}du\left| f^{\prime }(b)\right| ^{q} \end{array} \right) ^{\frac{1}{q}} \end{array} \right] . \end{aligned}$$

For the appearing integrals, we have

19 $$\begin{aligned} \int _{0}^{\frac{1}{2}}\frac{u^{\alpha }}{\left( ub+\left( 1-u\right) a\right) ^{2}}du&= {} \frac{1}{2^{\alpha +1}}\int _{0}^{1}\frac{u^{\alpha }}{ \left( \frac{u}{2}b+(1-\frac{u}{2})a\right) ^{2}}du \\&= {} \frac{1}{2^{\alpha +1}}\int _{0}^{1}\left( 1-v\right) ^{\alpha }\left( \frac{a+b}{2}\right) ^{-2}\left( 1-v\left( \frac{b-a}{b+a}\right) \right) ^{-2}du \\&= {} \frac{\left( a+b\right) ^{-2}}{2^{\alpha -1}\left( \alpha +1\right) } \begin{array}{c} _{2}F_{1}\left( 2,1;\alpha +2;\frac{b-a}{b+a}\right) \end{array} \\&= {} C_{3}\left( \alpha \right) , \end{aligned}$$

20 $$\begin{aligned} \int _{0}^{\frac{1}{2}}\frac{u^{\alpha +1}}{\left( ub+\left( 1-u\right) a\right) ^{2}}du&= {} \frac{1}{2^{\alpha +2}}\int _{0}^{1}\frac{u^{\alpha +1}}{ \left( \frac{u}{2}b+(1-\frac{u}{2})a\right) ^{2}}du \\&= {} \frac{1}{2^{\alpha +2}}\int _{0}^{1}\left( 1-v\right) ^{\alpha +1}\left( \frac{a+b}{2}\right) ^{-2}\left( 1-v\left( \frac{b-a}{b+a}\right) \right) ^{-2}du \\&= {} \frac{\left( a+b\right) ^{-2}}{2^{\alpha }\left( \alpha +2\right) } \begin{array}{c} _{2}F_{1}\left( 2,1;\alpha +3;\frac{b-a}{b+a}\right) \end{array} \\&= {} C_{4}\left( \alpha \right) , \end{aligned}$$

21 $$\begin{aligned} \int _{0}^{\frac{1}{2}}\frac{u^{\alpha }}{\left( ub+\left( 1-u\right) a\right) ^{2}}\left( 1-u\right) du&= {} C_{3}\left( \alpha \right) -C_{4}\left( \alpha \right) =C_{5}\left( \alpha \right) , \end{aligned}$$

22 $$\begin{aligned} \int _{\frac{1}{2}}^{1}\frac{\left( 1-u\right) ^{\alpha }}{\left( ub+\left( 1-u\right) a\right) ^{2}}du&= {} \int _{0}^{\frac{1}{2}}\frac{u^{\alpha }}{ \left( ua+\left( 1-u\right) b\right) ^{2}}du \\&= {} \frac{1}{2^{\alpha +1}}\int _{0}^{1}\frac{u^{\alpha }}{\left( \frac{u}{2} a+\left( 1-\frac{u}{2}\right) b\right) ^{2}}du \\&= {} \frac{1}{2^{\alpha +1}}\int _{0}^{1}u^{\alpha }b^{-2}\left( 1-\frac{u}{2} \left( 1-\frac{a}{b}\right) \right) ^{-2}du \\&= {} \frac{b^{-2}}{2^{\alpha +1}\left( \alpha +1\right) } \begin{array}{c} _{2}F_{1}\left( 2,\alpha +1;\alpha +2;\frac{1}{2}\left( 1-\frac{a}{b}\right) \right) \end{array} \\&= {} C_{6}\left( \alpha \right) , \end{aligned}$$

23 $$\begin{aligned} \int _{\frac{1}{2}}^{1}\frac{\left( 1-u\right) ^{\alpha }}{\left( ub+\left( 1-u\right) a\right) ^{2}}udu&= {} \int _{0}^{\frac{1}{2}}\frac{u^{\alpha }\left( 1-u\right) }{\left( ua+\left( 1-u\right) b\right) ^{2}}du \\&= {} \int _{0}^{\frac{1}{2}}\frac{u^{\alpha }}{\left( ua+\left( 1-u\right) b\right) ^{2}}du-\int _{0}^{\frac{1}{2}}\frac{u^{\alpha +1}}{\left( ua+\left( 1-u\right) b\right) ^{2}}du \\&= {} \frac{1}{2^{\alpha +1}}\int _{0}^{1}\frac{u^{\alpha }}{\left( \frac{u}{2} a+\left( 1-\frac{u}{2}\right) b\right) ^{2}}du \\&\quad-\frac{1}{2^{\alpha +2}}\int _{0}^{1}\frac{u^{\alpha +1}}{\left( \frac{u}{2} a+\left( 1-\frac{u}{2}\right) b\right) ^{2}}du \\&= {} \frac{1}{2^{\alpha +1}}\int _{0}^{1}u^{\alpha }b^{-2}\left( 1-\frac{u}{2} \left( 1-\frac{a}{b}\right) \right) ^{-2}du \\&\quad-\frac{1}{2^{\alpha +2}}\int _{0}^{1}u^{\alpha +1}b^{-2}\left( 1-\frac{u}{2} \left( 1-\frac{a}{b}\right) \right) ^{-2}du \\&= {} \left[ \begin{array}{c} \frac{b^{-2}}{2^{\alpha +1}\left( \alpha +1\right) } \begin{array}{c} _{2}F_{1}\left( 2,\alpha +1;\alpha +2;\frac{1}{2}\left( 1-\frac{a}{b}\right) \right) \end{array} \\ -\frac{b^{-2}}{2^{\alpha +2}\left( \alpha +2\right) } \begin{array}{c} _{2}F_{1}\left( 2,\alpha +2;\alpha +3;\frac{1}{2}\left( 1-\frac{a}{b}\right) \right) \end{array} \end{array} \right] \\&= {} C_{7}\left( \alpha \right) , \end{aligned}$$

24 $$\begin{aligned} \int _{\frac{1}{2}}^{1}\frac{\left( 1-u\right) ^{\alpha +1}}{\left( ub+\left( 1-u\right) a\right) ^{2}}du&= {} \int _{\frac{1}{2}}^{1}\frac{\left( 1-u\right) ^{\alpha }}{\left( ub+\left( 1-u\right) a\right) ^{2}}du-\int _{\frac{1}{2} }^{1}\frac{\left( 1-u\right) ^{\alpha }}{\left( ub+\left( 1-u\right) a\right) ^{2}}udu \\&= {} C_{6}\left( \alpha \right) -C_{7}\left( \alpha \right) =C_{8}\left( \alpha \right) . \end{aligned}$$

If we use (19–24) in (18) , we have (17). This completes the proof.

### **Corollary 2**

*In Theorem *7:

*(1) If we take *$\alpha =1$ w*e have the following Hermite–Hadamard–Fejer inequality for harmonically convex functions which is related to the left-hand side of (*5*):*

$$\begin{aligned}&\left| f\left( \frac{2ab}{a+b}\right) \int _{a}^{b}\frac{g\left( x\right) }{x^{2}}dx-\int _{a}^{b}\frac{f\left( x\right) g\left( x\right) }{ x^{2}}dx\right| \\&\quad \le \left\| g\right\| _{\infty }\left( b-a\right) ^{2}\left[ \begin{array}{c} C_{3}^{^{1-\frac{1}{q}}}\left( 1\right) \left[ \left( \begin{array}{c} C_{4}\left( 1\right) \left| f^{\prime }(a)\right| ^{q} \\ +C_{5}\left( 1\right) \left| f^{\prime }(b)\right| ^{q} \end{array} \right) \right] ^{\frac{1}{q}} \\ +C_{6}^{1-\frac{1}{q}}\left( 1\right) \left[ \left( \begin{array}{c} C_{7}\left( 1\right) \left| f^{\prime }(a)\right| ^{q} \\ +C_{8}\left( 1\right) \left| f^{\prime }(b)\right| ^{q} \end{array} \right) \right] ^{\frac{1}{q}} \end{array} \right] , \end{aligned}$$

*(2) If we take *$g\left( x\right) =1$*we have the following Hermite–Hadamard type inequality for harmonically convex functions in fractional integral forms which is related to the left-hand side of (*6*):*

$$\begin{aligned}&\left| f\left( \frac{2ab}{a+b}\right) -\frac{\varGamma \left( \alpha +1\right) }{2^{1-\alpha }}\left( \frac{ab}{b-a}\right) ^{\alpha }\left\{ \begin{array}{c} J_{\frac{a+b}{2ab}+}^{\alpha }\left( f\circ h\right) \left( 1/a\right) \\ +J_{\frac{a+b}{2ab}-}^{\alpha }\left( f\circ h\right) \left( 1/b\right) \end{array} \right\} \right| \\&\quad \le \frac{ab\left( b-a\right) }{2^{1-\alpha }}\left[ \begin{array}{l} C_{3}^{^{1-\frac{1}{q}}}\left( \alpha \right) \left[ \left( \begin{array}{c} C_{4}\left( \alpha \right) \left| f^{\prime }(a)\right| ^{q} \\ +C_{5}\left( \alpha \right) \left| f^{\prime }(b)\right| ^{q} \end{array} \right) \right] ^{\frac{1}{q}} \\ +C_{6}^{1-\frac{1}{q}}\left( \alpha \right) \left[ \left( \begin{array}{c} C_{7}\left( \alpha \right) \left| f^{\prime }(a)\right| ^{q} \\ +C_{8}\left( \alpha \right) \left| f^{\prime }(b)\right| ^{q} \end{array} \right) \right] ^{\frac{1}{q}} \end{array} \right] , \end{aligned}$$

(*3) If we take*$\alpha =1$*and *$g\left( x\right) =1$*we have the following Hermite–Hadamard type inequality for harmonically convex functions which is related to the left-hand side of (*4*):*

$$\begin{aligned}&\left| f\left( \frac{2ab}{a+b}\right) -\frac{ab}{b-a}\int _{a}^{b}\frac{ f\left( x\right) }{x^{2}}dx\right| \\&\quad \le ab\left( b-a\right) \left[ \begin{array}{l} C_{3}^{^{1-\frac{1}{q}}}\left( 1\right) \left[ \left( \begin{array}{c} C_{4}\left( 1\right) \left| f^{\prime }(a)\right| ^{q} \\ +C_{5}\left( 1\right) \left| f^{\prime }(b)\right| ^{q} \end{array} \right) \right] ^{\frac{1}{q}} \\ +C_{6}^{1-\frac{1}{q}}\left( 1\right) \left[ \left( \begin{array}{c} C_{7}\left( 1\right) \left| f^{\prime }(a)\right| ^{q} \\ +C_{8}\left( 1\right) \left| f^{\prime }(b)\right| ^{q} \end{array} \right) \right] ^{\frac{1}{q}} \end{array} \right] . \end{aligned}$$

We can state another inequality for $q>1$ as follows:

### **Theorem 8**

*Let*$f:I\subset \left( 0,\infty \right) \rightarrow \mathbb {R}$*be a differentiable function on*$I {{}^\circ }$, *the interior ofI*, *such that*$f^{\prime }\in L\left[ a,b\right]$, *where*$a,b\in I$. *If*$\left| f^{\prime }\right| ^{q},q>1,$*is harmonically convex* on $\left[ a,b\right]$, $g:\left[ a,b\right] \mathbb { \rightarrow R}$*is continuous and harmonically symmetric with respect to*$\frac{2ab}{a+b}$, *then the following inequality for fractional integrals holds:*

25 $$\begin{aligned}&\left| \begin{array}{c} \frac{f(a)+f(b)}{2}\left[ J_{1/b+}^{\alpha }\left( g\circ h\right) (1/a)+J_{1/a-}^{\alpha }\left( g\circ h\right) (1/b)\right] \\ -\left[ J_{1/b+}^{\alpha }\left( fg\circ h\right) (1/a)+J_{1/a-}^{\alpha }\left( fg\circ h\right) (1/b)\right] \end{array} \right| \\&\quad \le \frac{\left\| g\right\| _{\infty }ab\left( b-a\right) }{\varGamma (\alpha +1)}\left( \frac{b-a}{ab}\right) ^{\alpha } \\&\quad\quad \times \left[ \begin{array}{c} C_{9}^{\frac{1}{p}}\left( \alpha \right) \left[ \frac{\left| f^{\prime }(a)\right| ^{q}+3\left| f^{\prime }(b)\right| ^{q}}{8}\right] ^{ \frac{1}{q}} \\ +C_{10}^{\frac{1}{p}}\left( \alpha \right) \left[ \frac{3\left| f^{\prime }(a)\right| ^{q}+\left| f^{\prime }(b)\right| ^{q}}{8} \right] ^{\frac{1}{q}} \end{array} \right] \end{aligned}$$

*where*

$$\begin{aligned} C_{9}\left( \alpha \right)&= {} \frac{\left( a+b\right) ^{-2p}}{2^{\alpha p-2p+1}\left( \alpha p+1\right) } \begin{array}{c} _{2}F_{1}\left( 2p,1;\alpha p+2;\frac{b-a}{b+a}\right) \end{array} , \\ C_{10}\left( \alpha \right)&= {} \frac{b^{-2p}}{2^{\alpha p+1}\left( \alpha p+1\right) } \begin{array}{c} _{2}F_{1}\left( 2,\alpha p+1;\alpha p+2;\frac{1}{2}\left( 1-\frac{a}{b} \right) \right) \end{array} , \end{aligned}$$

*with*$$\alpha >1$$, $$h(x)=1/x$$, $$x\in \left[ \frac{1}{b},\frac{1}{a}\right]$$*and*$$1/p+1/q=1$$.

### *Proof*

Using (12), Hölder’s inequality and the harmonically convexity of $\left| f^{\prime }\right| ^{q}$, it follows that

26 $$\begin{aligned}&\left| \begin{array}{c} f\left( \frac{2ab}{a+b}\right) \left[ J_{\frac{a+b}{2ab}+}^{\alpha }\left( g\circ h\right) (1/a)+J_{\frac{a+b}{2ab}-}^{\alpha }\left( g\circ h\right) (1/b)\right] \\ -\left[ J_{\frac{a+b}{2ab}+}^{\alpha }\left( fg\circ h\right) (1/a)+J_{\frac{ a+b}{2ab}-}^{\alpha }\left( fg\circ h\right) (1/b)\right] \end{array} \right| \\&\quad \le \frac{\left\| g\right\| _{\infty }ab\left( b-a\right) }{\varGamma (\alpha +1)}\left( \frac{b-a}{ab}\right) ^{\alpha }\left[ \begin{array}{c} \int _{0}^{\frac{1}{2}}\frac{u^{\alpha }}{\left( ub+\left( 1-u\right) a\right) ^{2}}\left| f^{\prime }(\frac{ab}{ub+\left( 1-u\right) a} )\right| du \\ +\int _{\frac{1}{2}}^{1}\frac{\left( 1-u\right) ^{\alpha }}{\left( ub+\left( 1-u\right) a\right) ^{2}}\left| f^{\prime }(\frac{ab}{ub+\left( 1-u\right) a})\right| du \end{array} \right] \\&\quad \le \frac{\left\| g\right\| _{\infty }ab\left( b-a\right) }{\varGamma (\alpha +1)}\left( \frac{b-a}{ab}\right) ^{\alpha }\left[ \begin{array}{c} \left( \int _{0}^{\frac{1}{2}}\frac{u^{\alpha p}}{\left( ub+\left( 1-u\right) a\right) ^{2p}}du\right) ^{\frac{1}{p}} \\ \times \left( \int _{0}^{\frac{1}{2}}\left| f^{\prime }(\frac{ab}{ ub+(1-u)a})\right| ^{q}du\right) ^{\frac{1}{q}} \end{array} \right. \\&\quad \left. \begin{array}{c} +\left( \int _{\frac{1}{2}}^{1}\frac{\left( 1-u\right) ^{\alpha p}}{\left( ub+\left( 1-u\right) a\right) ^{2p}}du\right) ^{\frac{1}{p}} \\ \quad\times \left( \int _{\frac{1}{2}}^{1}\left| f^{\prime }(\frac{ab}{ ub+(1-u)a})\right| ^{q}du\right) ^{\frac{1}{q}} \end{array} \right] \\&\quad \le \frac{\left\| g\right\| _{\infty }ab\left( b-a\right) }{\varGamma (\alpha +1)}\left( \frac{b-a}{ab}\right) ^{\alpha }\left[ \begin{array}{c} \left( \int _{0}^{\frac{1}{2}}\frac{u^{\alpha p}}{\left( ub+\left( 1-u\right) a\right) ^{2p}}du\right) ^{\frac{1}{p}} \\ \times \left( \int _{0}^{\frac{1}{2}}u\left| f^{\prime }(a)\right| ^{q}+\left( 1-u\right) \left| f^{\prime }(b)\right| ^{q}du\right) ^{ \frac{1}{q}} \end{array} \right. \\&\quad \left. \begin{array}{c} +\left( \int _{\frac{1}{2}}^{1}\frac{\left( 1-u\right) ^{\alpha p}}{\left( ub+\left( 1-u\right) a\right) ^{2p}}du\right) ^{\frac{1}{p}} \\ \times \left( \int _{\frac{1}{2}}^{1}u\left| f^{\prime }(a)\right| ^{q}+\left( 1-u\right) \left| f^{\prime }(b)\right| ^{q}du\right) ^{ \frac{1}{q}} \end{array} \right] \\&\quad =\frac{\left\| g\right\| _{\infty }ab\left( b-a\right) }{\varGamma (\alpha +1)}\left( \frac{b-a}{ab}\right) ^{\alpha } \\&\quad \quad \times \left[ \left( \int _{0}^{\frac{1}{2}}\frac{u^{\alpha p}}{\left( ub+\left( 1-u\right) a\right) ^{2p}}du\right) ^{\frac{1}{p}}\left[ \frac{ \left| f^{\prime }(a)\right| ^{q}+3\left| f^{\prime }(b)\right| ^{q}}{8}\right] ^{\frac{1}{q}}\right. \\&\quad \quad \left. +\left( \int _{\frac{1}{2}}^{1}\frac{\left( 1-u\right) ^{\alpha p}}{ \left( ub+\left( 1-u\right) a\right) ^{2p}}du\right) ^{\frac{1}{p}}\left[ \frac{3\left| f^{\prime }(a)\right| ^{q}+\left| f^{\prime }(b)\right| ^{q}}{8}\right] ^{\frac{1}{q}}\right] . \end{aligned}$$

For the appearing integrals, we have

27 $$\begin{aligned} \int _{0}^{\frac{1}{2}}\frac{u^{\alpha p}}{\left( ub+\left( 1-u\right) a\right) ^{2p}}du&= {} \frac{1}{2^{\alpha p+1}}\int _{0}^{1}\frac{u^{\alpha p}}{ \left( \frac{u}{2}b+(1-\frac{u}{2})a\right) ^{2p}}du \\&= {} \frac{1}{2^{\alpha p+1}}\int _{0}^{1}\left( 1-v\right) ^{\alpha p}\left( \frac{a+b}{2}\right) ^{-2p}\left[ 1-v\left( \frac{b-a}{b+a}\right) \right] ^{-2p}dv \\&= {} \frac{\left( a+b\right) ^{-2p}}{2^{\alpha p-2p+1}\left( \alpha p+1\right) } \begin{array}{c} _{2}F_{1}\left( 2p,1;\alpha p+2;\frac{b-a}{b+a}\right) \end{array} \\&= {} C_{9}\left( \alpha \right) . \end{aligned}$$

Similarly, we have

28 $$\begin{aligned} \int _{\frac{1}{2}}^{1}\frac{\left( 1-u\right) ^{\alpha p}}{\left( ub+\left( 1-u\right) a\right) ^{2p}}du&= {} \int _{0}^{\frac{1}{2}}\frac{u^{\alpha p}}{ \left( ua+\left( 1-u\right) b\right) ^{2p}}du \\&= {} \frac{1}{2^{\alpha p+1}}\int _{0}^{1}\frac{u^{\alpha p}}{\left( \frac{u}{2} a+\left( 1-\frac{u}{2}\right) b\right) ^{2p}}du \\&= {} \frac{1}{2^{\alpha p+1}}\int _{0}^{1}u^{\alpha }b^{-2p}\left( 1-\frac{u}{2} \left( 1-\frac{a}{b}\right) \right) ^{-2p}du \\&= {} \frac{b^{-2p}}{2^{\alpha p+1}\left( \alpha p+1\right) } \begin{array}{c} _{2}F_{1}\left( 2,\alpha p+1;\alpha p+2;\frac{1}{2}\left( 1-\frac{a}{b} \right) \right) \end{array} \\&= {} C_{10}\left( \alpha \right) . \end{aligned}$$

If we use (27) and (28) in (26), we have (25). This completes the proof.

### **Corollary 3**

*In Theorem* 8*:*

*(1) If we take*$\alpha =1$*we have the following Hermite–Hadamard–Fejer inequality for harmonically convex functions which is related to the left-hand side of* (5):

$$\begin{aligned}&\left| f\left( \frac{2ab}{a+b}\right) \int _{a}^{b}\frac{g\left( x\right) }{x^{2}}dx-\int _{a}^{b}\frac{f\left( x\right) g\left( x\right) }{ x^{2}}dx\right| \\&\quad \le \left\| g\right\| _{\infty }\left( b-a\right) ^{2}\left[ \begin{array}{c} C_{9}^{\frac{1}{p}}\left( 1\right) \left[ \frac{\left| f^{\prime }(a)\right| ^{q}+3\left| f^{\prime }(b)\right| ^{q}}{8}\right] ^{ \frac{1}{q}} \\ +C_{10}^{\frac{1}{p}}\left( 1\right) \left[ \frac{3\left| f^{\prime }(a)\right| ^{q}+\left| f^{\prime }(b)\right| ^{q}}{8}\right] ^{ \frac{1}{q}} \end{array} \right] , \end{aligned}$$

*(2) If we take*$g\left( x\right) =1$*we have following Hermite–Hadamard type inequality for harmonically convex functions in fractional integral forms which is related to the left-hand side of* (6):

$$\begin{aligned}&\left| f\left( \frac{2ab}{a+b}\right) -\frac{\varGamma \left( \alpha +1\right) }{2^{1-\alpha }}\left( \frac{ab}{b-a}\right) ^{\alpha }\left\{ \begin{array}{c} J_{\frac{a+b}{2ab}+}^{\alpha }\left( f\circ h\right) \left( 1/a\right) \\ +J_{\frac{a+b}{2ab}-}^{\alpha }\left( f\circ h\right) \left( 1/b\right) \end{array} \right\} \right| \\&\quad \le \frac{ab\left( b-a\right) }{2^{1-\alpha }}\left[ \begin{array}{c} C_{9}^{\frac{1}{p}}\left( \alpha \right) \left[ \frac{\left| f^{\prime }(a)\right| ^{q}+3\left| f^{\prime }(b)\right| ^{q}}{8}\right] ^{ \frac{1}{q}} \\ +C_{10}^{\frac{1}{p}}\left( \alpha \right) \left[ \frac{3\left| f^{\prime }(a)\right| ^{q}+\left| f^{\prime }(b)\right| ^{q}}{8} \right] ^{\frac{1}{q}} \end{array} \right] , \end{aligned}$$

*(3) If we take*$\alpha =1$*and*$g\left( x\right) =1$*we have the following Hermite–Hadamard type inequality for harmonically convex functions which is related to the left-hand side of* (4):

$$\begin{aligned}&\left| f\left( \frac{2ab}{a+b}\right) -\frac{ab}{b-a}\int _{a}^{b}\frac{ f\left( x\right) }{x^{2}}dx\right| \\&\quad \le ab\left( b-a\right) \left[ \begin{array}{l} C_{9}^{\frac{1}{p}}\left( 1\right) \left[ \frac{\left| f^{\prime }(a)\right| ^{q}+3\left| f^{\prime }(b)\right| ^{q}}{8}\right] ^{ \frac{1}{q}} \\ +C_{10}^{\frac{1}{p}}\left( 1\right) \left[ \frac{3\left| f^{\prime }(a)\right| ^{q}+\left| f^{\prime }(b)\right| ^{q}}{8}\right] ^{ \frac{1}{q}} \end{array} \right] . \end{aligned}$$

## Conclusion

In this paper, new Hermite–Hadamard type inequalities for harmonically convex functions in fractional integral forms are given and Hermite–Hadamard–Fejer inequalities for harmonically convex functions in fractional integral forms are built. Also, an integral identity and some Hermite–Hadamard–Fejer type integral inequalities for harmonically convex functions in fractional integral forms are obtained.
